# Hydration of *N*-Hydroxyurea from Ab Initio Molecular Dynamics Simulations

**DOI:** 10.3390/molecules29112435

**Published:** 2024-05-22

**Authors:** Mateusz Balicki, Maciej Śmiechowski

**Affiliations:** Department of Physical Chemistry, Faculty of Chemistry, Gdańsk University of Technology, Narutowicza 11/12, 80-233 Gdańsk, Poland; mateuszbalicki@outlook.com

**Keywords:** ab initio molecular dynamics, density functional theory, hydration, *N*-hydroxyurea

## Abstract

*N*-Hydroxyurea (HU) is an important chemotherapeutic agent used as a first-line treatment in conditions such as sickle cell disease and β-thalassemia, among others. To date, its properties as a hydrated molecule in the blood plasma or cytoplasm are dramatically understudied, although they may be crucial to the binding of HU to the radical catalytic site of ribonucleotide reductase, its molecular target. The purpose of this work is the comprehensive exploration of HU hydration. The topic is studied using ab initio molecular dynamic (AIMD) simulations that apply a first principles representation of the electron density of the system. This allows for the calculation of infrared spectra, which may be decomposed spatially to better capture the spectral signatures of solute–solvent interactions. The studied molecule is found to be strongly hydrated and tightly bound to the first shell water molecules. The analysis of the distance-dependent spectra of HU shows that the *E* and *Z* conformers spectrally affect, on average, 3.4 and 2.5 of the closest H_2_O molecules, respectively, in spheres of radii of 3.7 Å and 3.5 Å, respectively. The distance-dependent spectra corresponding to these cutoff radii show increased absorbance in the red-shifted part of the water OH stretching vibration band, indicating local enhancement of the solvent’s hydrogen bond network. The radially resolved IR spectra also demonstrate that HU effortlessly incorporates into the hydrogen bond network of water and has an enhancing effect on this network. Metadynamics simulations based on AIMD methodology provide a picture of the conformational equilibria of HU in solution. Contrary to previous investigations of an isolated HU molecule in the gas phase, the *Z* conformer of HU is found here to be more stable by 17.4 kJ·mol^−1^ than the *E* conformer, pointing at the crucial role that hydration plays in determining the conformational stability of solutes. The potential energy surface for the OH group rotation in HU indicates that there is no intramolecular hydrogen bond in *Z*-HU in water, in stark contrast to the isolated solute in the gas phase. Instead, the preferred orientation of the hydroxyl group is perpendicular to the molecular plane of the solute. In view of the known chaotropic effect of urea and its *N*-alkyl-substituted derivatives, *N*-hydroxyurea emerges as a unique urea derivative that exhibits a kosmotropic ordering of nearby water. This property may be of crucial importance for its binding to the catalytic site of ribonucleotide reductase with a concomitant displacement of a water molecule.

## 1. Introduction

Osmolytes are small organic solutes that influence the stability of the native structure of proteins in solution [1,2,3,4]. Their role is intimately coupled with the role of hydration water, which is now known to actively shape the native structure of proteins [5]. Protein hydration shells are important for their functioning, and phenomena such as protein aggregation and ligand binding are also mediated to a large extent by the hydration water [5]. The compatibility of osmolyte and protein hydration shells is a crucial factor in predicting the stabilizing or destabilizing nature of a particular cosolvent [3]. Notably, urea and *N*-alkyl-substituted ureas are important as potent destabilizing agents that bind directly to model peptides and disrupt their hydration shell [2,6].

*N*-hydroxyurea (HU) is an example of an important *N*-substituted urea that, instead of an alkyl group, has a hydroxyl group introduced, thus forming a hydroxamic group in the molecule. HU is an important chemotherapeutic agent that shows antineoplastic action in myeloproliferative disorders, meningiomas, breast cancer, etc. [7,8,9]. Nowadays, it is the recommended first-line treatment for sickle cell disease; it has been used for this indication since the late 1980s and was officially approved in 1997 in the USA and 2007 in the EU [10,11,12]. HU is also administered in β-thalassemia, where it significantly reduces the need for blood transfusions in affected patients [13,14]. It may also be applied to selected skin conditions, e.g., psoriasis [15,16]. However, the prolonged therapeutic use of HU can lead to serious side effects, including various cutaneous conditions, nonmelanoma skin cancers, general bone marrow suppression, reduced spermatogenesis, and gastrointestinal effects, and strict monitoring of the patient during treatment is advised [13,15,17].

The molecular mechanism of action of HU is well known today [18,19,20,21,22]. Hydroxyurea acts as an inhibitor of DNA replication by affecting the activity of ribonucleotide reductase (RNR). This enzyme is a catalyst for the reduction of ribonucleoside diphosphates to the corresponding deoxyribonucleotides. Notably, it is one of the few proteins that fully harness the power of free radical reactions in vivo. A stable hydroxylated tyrosyl radical—residue Tyr-176 in the human protein—is a prominent and conserved feature of RNR and serves as the target for HU acting as a radical scavenger [18,20]. The binding of HU and its oxidized product at the catalytic site were confirmed by docking studies supported by molecular dynamics simulations [20]. Notably, the active state of the catalytic site is stabilized by interactions of the key tyrosine radical with neighboring Asp and Lys residues mediated by a single water molecule that is strongly hydrogen bonded to the radical site [18]. In an unrelated mechanism, HU also binds to oxy-hemoglobin, producing its nitroxide radical, which ultimately leads to the release of nitric oxide [22].

Hydroxyurea also forms stable dimers that, thus far, have been studied computationally in the gas phase and experimentally by matrix isolation infrared spectroscopy [23,24]. The interaction energy between the two HU subunits may be up to 60 kJ·mol^−1^ [24], but, surprisingly, the strongest bound dimer structures are not the ones that can be experimentally isolated in an Ar matrix and that exhibit binding energies close to half of the energy of the most stable dimer [23]. The implications of the dimer formation for HU toxicity, however, have not been studied thus far.

**Figure 1:**
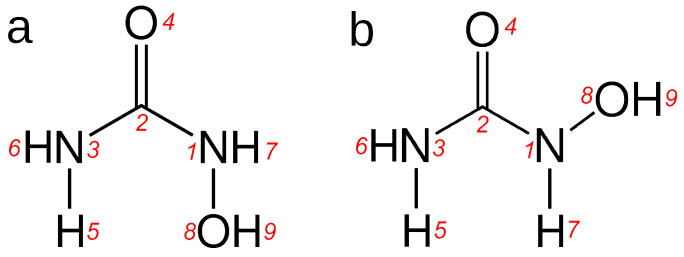
The studied conformers of hydroxyurea: (**a**) *E*-hydroxyurea; (**b**) *Z*-hydroxyurea. Italicized numbers indicate the atom numbering scheme used in this work.

Despite its importance in current medicine, HU is understudied when it comes to the mode of its interactions in water. As a chemotherapeutic present in blood plasma, knowledge of its hydration may be important for discussing its stability and transport in cells. In view of the importance of stabilization of the radical site in RNR mediated by a water molecule, as discussed above, the hydration properties of HU may have important implications for the substitution of said water molecule by HU as a first step to enzyme inactivation. Thus far, only microhydrated HU clusters with water have been studied computationally using quantum chemistry methods [25]. The *N*-hydroxyurea molecule is known in two principal conformers, *E*-HU and *Z*-HU; see Figure 1. The relative stability of the two in the gas phase is relatively well known [26,27,28,29]. These studies unanimously point to *E*-HU as being the more stable conformer. It is also the one that is present in crystalline *N*-hydroxyurea [30,31]. Strong hydration of HU was indirectly suspected based on volumetric data on its aqueous solutions [32]. However, no comprehensive computational study of hydrated HU has been attempted thus far. In particular, no previous molecular dynamic (MD) simulations seem to have been performed for pure aqueous HU solutions in terms of revealing its hydration structure, although previous MD studies of HU docking to its molecular targets were performed [20,22]. In this respect, the present contribution delivers important knowledge about the structure and spectral properties of two conformers of *N*-hydroxyurea, thus filling an important gap in the research on this medically important compound.

In this work, ab initio molecular dynamic (AIMD) simulations [33] based on density functional theory (DFT) [34,35] were applied to study the hydration of HU conformers in a relatively large system composed of an HU molecule solvated by 109 water molecules. The first principles treatment of the electron density of the system allows for the study of mutual polarization effects between the species, which is crucial for a deeper understanding of the infrared (IR) spectrum of the system [36,37,38,39]. While these can be in principle accounted for in polarizable and/or reactive force fields, the DFT picture relies fully on the electron density of the system and is therefore less dependent on arbitrary assumptions. The spectral decomposition techniques introduced for pure liquids [36,38] and extended towards binary solute–solvent systems [37,39], which are leveraged in this work, provide unique interpretational power that allows for deciphering the spatial and temporal correlations that ultimately underlie the experimentally accessible IR spectra of liquid systems. The revised Perdew–Burke–Ernzerhof (revPBE) density functional [40,41] was chosen as it provides excellent reproduction of numerous static and dynamic properties of liquid water and aqueous ionic solutions [42,43,44].

The research hypothesis posed in this work is that HU is a strongly hydrated solute and that solvation by water has a non-negligible influence on its conformation. Additionally, its strong interactions with water may have important implications for binding at the radical catalytic site of RNR enzymes. Sophisticated computational infrared spectral analysis and free energy landscape exploration techniques, described in detail below, were applied to positively verify this hypothesis and conclusively prove that HU indeed shows an enhancing effect on the hydrogen bond network of water, at variance to the *N*-alkyl-substituted ureas.

## 2. Theoretical Background

Using linear response theory, the linear absorption coefficient of a system is obtained as a Fourier transform (FT) of the total dipole moment time correlation function [45,46,47],

(1) $$\alpha \left(\omega \right)=F\left(\omega \right)\int_{-\infty }^{\infty }dt\;e^{-i\omega t}\langle M(0)M(t)\rangle \;,$$

where $\langle \ldots \rangle$ is an ensemble average and F(ω) is the quantum correction prefactor time correlation function of the classical total dipole moment [46]. Using the maximally localized Wannier functions (MLWFs) formalism [48], it is possible to formally decompose the total dipole moment to discrete molecular dipole moments, i.e., $M=\sum_{i=1}^{N}\mu_{i}$. The latter can then be obtained classically by summing over positive nuclei and negative MLWF centers within a molecule, where in a closed-shell system, each center has a formal charge of −2e.

The analysis of the computational IR spectra of a solute–solvent system is greatly enhanced by using spatial decomposition techniques that allow for the disentanglement of the underlying solute and solvent contributions, as well as the mutual couplings between the two. Radially resolved IR spectra, initially introduced for liquid water [36], but later successfully applied to other pure liquids [38] and extended to cover also solute–solvent systems [37,39], enable the identification of the solvation shell contributions to the IR spectra of the studied aqueous solutions. Although a detailed derivation is available in the above-cited works, the methodology is briefly introduced below. The principal idea is based on the decomposition of the total dipole moment of the system on a regular spatial grid using Gaussian smoothing, as

(2) $$\rho_{\mu }\left(t,r\right)=\sum_{i=1}^{N}\mu_{i}\left(t\right)\frac{1}{{\left(2\pi \sigma^{2}\right)}^{3/2}}\exp\left[-\frac{{\left(R_{i}\left(t\right)-r\right)}^{2}}{2\sigma^{2}}\right]\;,$$

where $\rho_{\mu }$ is the molecular dipole moment density at time *t* and grid point r, $\mu_{i}$ is the instantaneous molecular dipole moments, $R_{i}$ is the current center of mass positions of the molecules, σ is the standard deviation of the three-dimensional Gaussian, and the summation runs over the *N* molecules making up the system. By fixing the center of mass of the solute at the origin, the cross correlation between the solute’s dipole moment and the dipole moment density gives rise to the radially resolved IR spectrum, as

(3) $$\alpha_{\times }\left(\omega ,r\right)=F\left(\omega \right)4\pi r^{2}\int_{-\infty }^{\infty }dt\;e^{-i\omega t}\mu_{s}\left(0\right)\rho_{\mu }\left(t,r\right)\;,$$

where F(ω) is the above-mentioned quantum correction prefactor [46] and the $4\pi r^{2}$ factor results from integrating out the angular dependence in the radial shell. The IR absorption coefficient thus defined effectively disentangles the spectra at each frequency in terms of solvation shell contributions to the total IR spectrum.

Another decomposition scheme previously proposed for solute–solvent systems [37,39] and originally applied to bulk water [36] explores the possibility to calculate the dipole moment within a smoothly carved spherical region around the solute as

(4) $$\mu_{s}^{R}\left(t\right)=N_{s}^{R}\left(t\right)\left(\mu_{s}\left(t\right)+\sum_{i}^{\mathrm{solvent}}P_{s,i}\left(t\right)\mu_{i}\left(t\right)\right)\;,$$

where the logistic function $P_{s,i}\left(t\right)=\left\{1+\exp\left[\left(R_{s,i}\left(t\right)-R_{c}\right)D^{-1}\right]\right\}$ ensures a gradual scaling of the molecular dipole of *i*th water molecule at a distance $R_{s,i}$ from the solute when transitioning the cutoff radius $R_{c}$ with the parameter *D* controlling the width of the transitional region. The distance-dependent dipole moment is normalized to a single molecule by the factor $N_{s}^{R}\left(t\right)={\left[1+\sum_{i}P_{s,i}^{2}\left(t\right)\right]}^{-0.5}$. By Fourier transforming its time correlation function, the distance-dependent IR absorption coefficient is defined as

(5) $$\alpha_{s}^{R}\left(\omega ,R_{c}\right)=F\left(\omega \right)\int_{-\infty }^{\infty }dt\;e^{-i\omega t}\mu_{s}^{R}\left(0\right)\mu_{s}^{R}\left(t\right)\;.$$

By considering cutoff radii $R_{c}$ that are on the order of the solvation shell separation from the solute, only the IR spectrum of the solute and its neighboring, most spectrally affected solvent molecules may be effectively selected. This approach is conceptually very similar to the spectral decomposition methods applied to experimental IR spectra that provide the route to extracting the solute-affected IR spectrum of water [49].

Finally, in order to explore the free energy surface for the transition between the *E*-HU and *Z*-HU conformers, well-tempered metadynamics (WT-MTD) simulations are applied [50,51,52]. Metadynamics is a non-equilibrium sampling technique that allows one to probe the free energy landscape along the selected collective variable (CV) s by introducing a bias potential of the form

(6) $$V_{\mathrm{bias}}\left(s,t\right)=\sum_{t^{\prime }=k\tau_{G}}^{t^{\prime }<t}W\exp\left(-\frac{{\left(s\left(t\right)-s^{\prime }\right)}^{2}}{2\sigma^{2}}\right)\;,$$

where Gaussian repulsive potentials of width σ and height *W* are deposited in regular intervals $\tau_{G}$ along the CV, with $s^{\prime }$ denoting the previous values of the CV. The time limit of the bias potential then provides an unbiased estimate of the underlying free energy surface as

(7) $$V_{\mathrm{bias}}\left(s,t\to \infty \right)=-F\left(s\right)+C\;,$$

where *C* is an additive constant that is irrelevant to discussion of free energy differences.

In the WT-MTD modification, the Gaussian height becomes history dependent via

(8) $$W\left(t^{\prime }\right)=W_{0}\exp\left(-\frac{V(s^{\prime },t^{\prime })}{k_{B}T_{\mathrm{bias}}}\right)\;,$$

where $T_{\mathrm{bias}}$ is the biasing temperature, which is typically several times higher than the actual system temperature. The reduction in the Gaussian height with increasing simulation time ensures a smoother reconstruction of the free energy surface.

## 3. Results and Discussion

**Figure 2:**
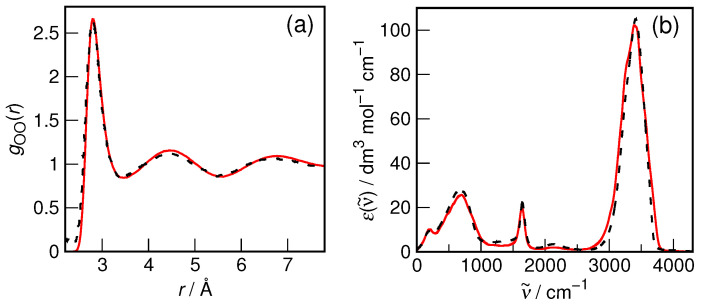
(**a**) Oxygen–oxygen radial distribution function of liquid water from AIMD simulations (red) and from X-ray diffraction measurements [56] (black). (**b**) Infrared absorption spectrum of liquid water on the decadic molar absorptivity scale from AIMD simulations (red) and from spectroscopic measurements [57] (black).

Although the accuracy of the revPBE/TZV2P-D3 method in describing liquid water structure and dynamics is well established [42,53,54,55], the most important parameters relevant to the present study, i.e., oxygen–oxygen radial distribution function and IR spectrum, were rechecked to confirm its applicability. The results for the bulk water system are shown in Figure 2. It is evident that both the static properties (as exemplified by RDF) and the dynamic ones (as represented by the IR spectrum) are indeed reproduced with formidable accuracy in the simulations as compared to the recommended experimental data. Therefore, there is reasonable confidence that the current computational protocol is fully adequate to study the hydration of small organic solutes.

**Figure 3:**
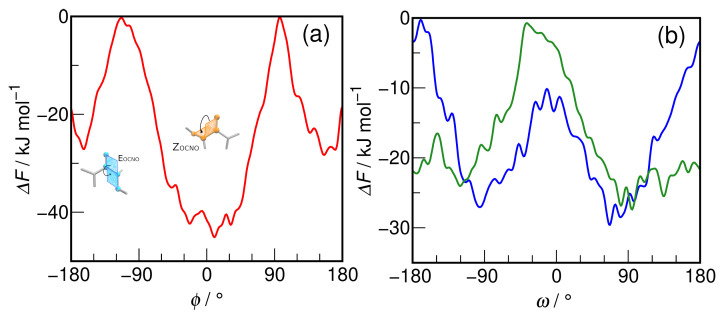
Free energy profiles for the rotation around (**a**) OCNO dihedral angle (ϕ) in hydroxyurea (red) and (**b**) CNOH dihedral angle (ω) in *E*-hydroxyurea (green) and *Z*-hydroxyurea (blue).

Before describing in detail the hydration shell of HU, the WT-MTD results are first analyzed in order to check the free energy surface for interconversion of both conformers (cf. Figure 3). The average one-dimensional free energy profile for the rotation of the OCNO dihedral angle (ϕ) is shown in Figure 3a. It is obvious that the WT-MTD simulation unequivocally points at *Z*-HU as the more stable conformer in an aqueous solution. The free energy minimum for *Z*-HU is located at 10°, which is sufficiently close to 0 to state that the OCNO atoms are almost coplanar. In turn, the minima for *E*-HU are located at +156° and −163°, so the preferred arrangement of the OCNO group is not coplanar but tilted so that the O atom of hydroxy group forms a ±20° angle with the OCN plane of *E*-HU. The two free energy minima have almost the same depth, and the average ΔF for *E*-HU ⇄ *Z*-HU interconversion is 17.4 kJ·mol^−1^. The estimated equilibrium constant value for this reaction is K=1118, so *Z*-HU can be expected to be the dominant form of HU in an aqueous solution. However, the conformers are separated by a sizable free energy barrier for the interconversion, ∼45 kJ·mol^−1^ in the *Z*-HU → *E*-HU direction and ∼28 kJ·mol^−1^ in the opposite direction. Since the height of the barrier from the *E*-HU site is approx. $11k_{B}T$, the *E* conformer may be trapped in its free energy minimum, and the interconversion reaction is a rare event. This is an important consideration in view of the fact that hydroxyurea exhibits the *E* configuration in the crystal [30,31].

Hydration has a profound impact on the relative stability of hydroxyurea conformers. In the gas phase, the *E* conformer is usually found to be the more stable form. The free energy (ΔG) difference is reported as 11.7 kJ·mol^−1^ by Remko et al. [28] and 8.3 kJ·mol^−1^ by Sałdyka [26] at the B3LYP/6-311+G(d,p) and MP2/6-311++G(2d,2p) level of theory, respectively. Di Gregorio et al. report an energy difference of 9.5 kJ·mol^−1^ at the MP2/aug-cc-pVDZ level [27], while La Manna and Barone provide the value of 13.1 kJ·molmol^−1^ at the MP2/6-31G** level [29]. Concomitantly, hydroxyurea crystallizes solely as *E*-HU [30,31]. The reported AIMD simulations deliver the opposite order of relative stability of conformers, pointing to the importance of hydration effects in determining the preferred conformation of small solutes in an aqueous solution. It is also worth noting that the gas phase energy barrier for *E*-HU ⇄ *Z*-HU interconversion has the opposite order of barrier heights than found here, namely 41.5 kJ·mol^−1^ from the *E*-HU direction and 33.2 kJ·mol^−1^ from the *Z*-HU direction [26]. This is, however, understandable considering that both conformers have different stabilities in an aqueous solution than they do in the gas phase.

In addition to the main dihedral angle of the backbone of HU, the free energy surface for the rotation of the hydroxyl hydrogen atom around the CNOH dihedral of urea was also investigated separately for each conformer. The one-dimensional free energy profiles for the rotation of the CNOH dihedral angle (ω) are shown in Figure 3b. In *E*-hydroxyurea, a large free energy barrier is noticeable around 0° (24–27 kJ·mol^−1^), so that the conformation in which the O-H bond point towards the nitrogen atom of the NH_2_ group is highly unlikely. Outside this barrier, the free energy surface is fairly flat, indicating an unhindered rotation of the O-H bond in the angular range of 90°–180°. On the contrary, in *Z*-HU, two free energy barriers are found: one at 0° (17–19 kJ·mol^−1^) and another larger one at ∼180° (27–29 kJ·mol^−1^). The *Z* conformer also shows two well-defined minima at ca. ±90°, indicating that the preferred conformation is with the O-H bond rotated perpendicularly to the OCNO coplanar arrangement.

The free energy barriers around ω=0° are well explained by steric clashes. In the case of *Z*-HU, the O-H bond would be rotated towards the carbonyl oxygen, and even though an intramolecular hydrogen bond (H-bond) may potentially form, the intramolecular distance between the two oxygen atoms is just 2.52 Å and the linear O-H···O arrangement is highly unlikely given the tetrahedral N–O–H angle, which is close to 109.5°. The possible H-bond would thus be very strained and weak. Likewise, in *E*-HU the hydroxyl hydrogen atom would point towards the H atom of the NH_2_ group, making such conformations energetically very unfavorable. The two HU conformers differ, however, in respect to the most probable ω value. While *E*-HU exhibits a relatively flat free energy surface outside the barrier at 0°, *Z*-HU clearly shows a preference for ω=±90°, i.e., perpendicular to the plane specified by the OCNO atoms.

**Figure 4:**
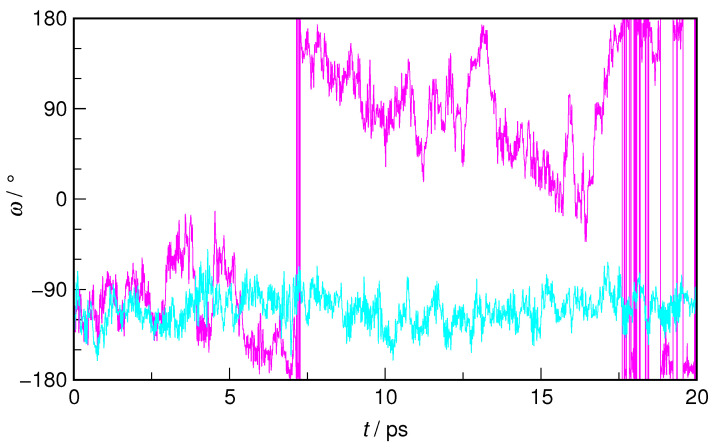
The time evolution of the CNOH dihedral angle (ω) in *E*-hydroxyurea during a selected microcanonical equilibrium AIMD trajectory (cyan) and during the well-tempered metadynamics trajectory, with ω as the collective variable (magenta).

In order to illustrate the exploration of the potential energy surface corresponding to a selected collective variable, the time evolution of the ω dihedral angle in *E*-HU during the WT-MTD run is shown in Figure 4 and juxtaposed with its changes in an equilibrium AIMD trajectory. It is readily visible that, while in the latter, the conformation of *E*-HU is described by ω values in the −160°–60° range (−110° on average), in WT-MTD, the system, during its trajectory, also explores the energetically unfavorable region around 0°, where the free energy barrier is located.

The rotation of the hydroxyl group was also extensively studied, however, only for isolated HU in the gas phase. In both MP2 and DFT calculations, the *E*-HU conformer with ω≈120° was found to be the most stable one, with a secondary energetic minimum at ω≈70°, the two being separated by a free energy barrier of ca. 11 kJ·mol^−1^ [26,27,28]. We also note that ω≈100° is found in *E*-hydroxyurea crystals [30,31]. In an aqueous solution, no discrete minima are observed, but rather no clear preference for a well-defined ω value, suggesting that hydration of the hydroxyl group has an impact on its rotation in the HU molecule. The most striking difference is observed, however, for the *Z*-HU conformer. While gas phase calculations unanimously point to the stabilizing role of the weak intramolecular H-bond [26,27,28], a clear preference is found for ω=±90° in *Z*-HU. As demonstrated below, the loss of the weak intramolecular H-bond is more than compensated for in an aqueous solution by the formation of H-bonds to the hydrating water molecules.

**Figure 5:**
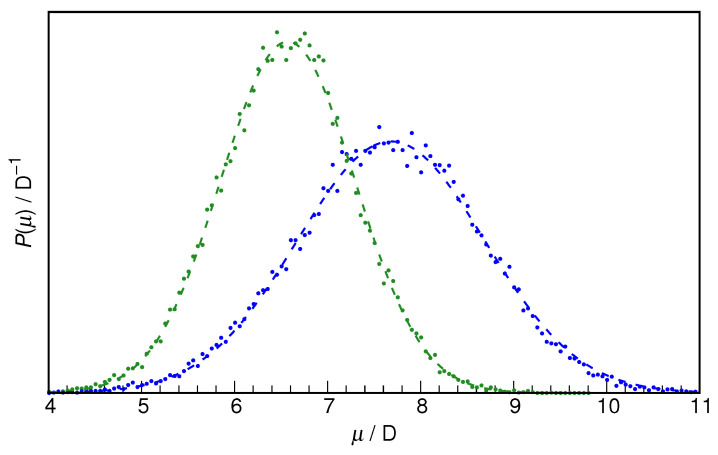
Normalized distribution functions of molecular dipole moments of *E*-hydroxyurea (green dots) and *Z*-hydroxyurea (blue dots) along with the respective fits to normal distribution (dashed lines).

In search for the possible reasons for the different hydration properties of the two studied HU isomers, the molecular dipole moment of HU from equilibrium AIMD simulations is first examined. As seen in Figure 5, the molecular dipole moments are characterized by normal distributions, as is typical of molecules possessing a permanent dipole moment in the liquid phase [38]. Due to charge redistribution in *Z*-HU, it shows a more than 1 D higher dipole moment than the *E*-HU conformer (7.68 D and 6.57 D, respectively). One can anticipate that the more polar solute will be better hydrated by water; however, this may lead to oversimplification, as, for example, dimethyl sulfoxide and dimethyl sulfone have almost the same dipole moments in an aqueous solution (7.36 D and 7.23 D, respectively), but the latter shows a dramatically weakened hydration sphere [58].

**Figure 6:**
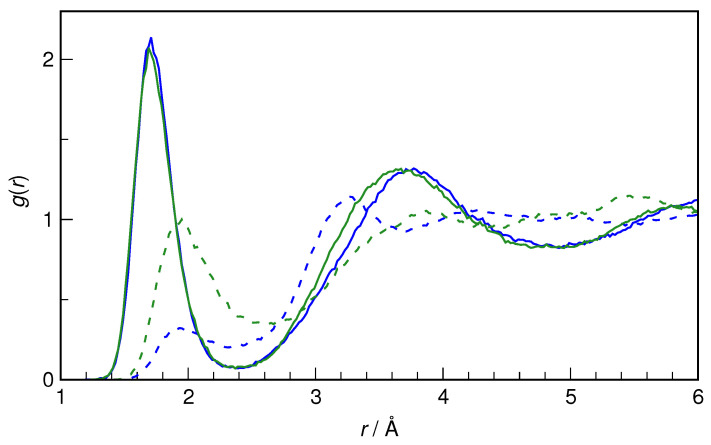
Radial distribution functions for H_9_···O*_w_* pairs (solid lines) and O_8_···H*_w_* pairs (dashed lines) in *E*-hydroxyurea (green) and *Z*-hydroxyurea (blue).

Both conformers of HU show several hydration sites that can interact favorably with water (i.e., the C=O group, the NH_2_ group, and the hydroxamic group, NHOH). It was found from previous ab initio studies of HU microhydration by a single H_2_O molecule that the configurations with H_2_O donating an H-bond to carbonyl oxygen have very similar interaction energies to ones in which the hydroxyl group of HU donates an H-bond to water, irrespective of the HU conformer [25]. The latter type of interaction is compared first for both conformers of HU; see Figure 6. The hydroxyl group of HU is seen to be a very good proton donor in an H-bond. The nearest H_9_···O*_w_* distance is 1.7 Å on average (for both conformers), and the first peak of radial distribution functions integrates to 1.0 in each case, confirming the status of the OH group of HU as a strong proton donor.

Although it is an excellent proton donor, the hydroxyl group is a rather weak proton acceptor, which is also seen in Figure 6. Here, the differences between the conformers become noticeable. Although the nearest O_8_···H*_w_* distance is still the same (1.94 Å), the integration over the first peak gives a value of 0.5 for *Z*-HU and 1.1 for *E*-HU. The H-bonds donated by H_2_O to O_8_ are weaker than those accepted by water from the OH group, as indicated by the interatomic distance being larger by 0.024 Å. Also, while *E*-HU O_8_ accepts on average a single H-bond from water, for *Z*-HU, such hydrogen bonds are formed only transiently, existing for about half of the trajectory time. Steric factors are the most probable cause of this difference between the two conformers.

**Figure 7:**
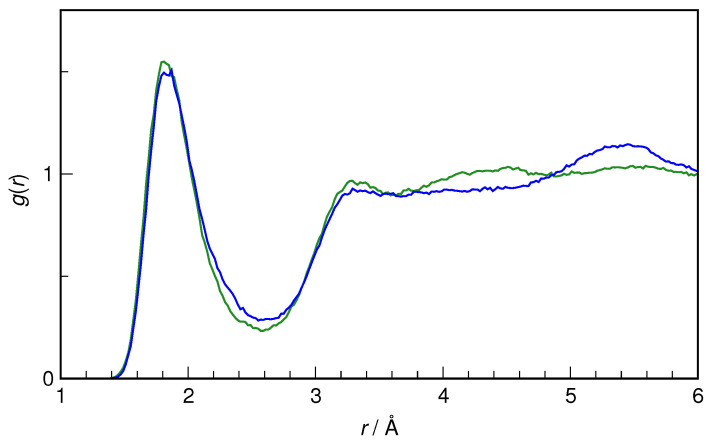
Radial distribution functions for O_4_···H*_w_* pairs in *E*-hydroxyurea (green) and *Z*-hydroxyurea (blue).

The hydration of the carbonyl oxygen of HU (O_4_) is examined next. As shown in Figure 7, even though the intramolecular surroundings of O_4_ are very different in *E*-HU vs. *Z*-HU, the hydrogen bonding picture is roughly the same. The most probable O_4_···H*_w_* distance is ca. 1.8 Å in both cases, and the integration over the first peak in RDF gives a similar value of coordination number (2.5 and 2.6 for *E*-HU and *Z*-HU, respectively). Thus, the carbonyl oxygen is a decent H-bond acceptor in both conformers. It is noted here that, in unsubstituted urea, the carbonyl oxygen coordination number (by water H atoms) is also 2.6, with a very similar first maximum value in RDF, indicating that the substitution by a hydroxyl group does not noticeably alter the hydration shell of O_4_ [6].

**Figure 8:**
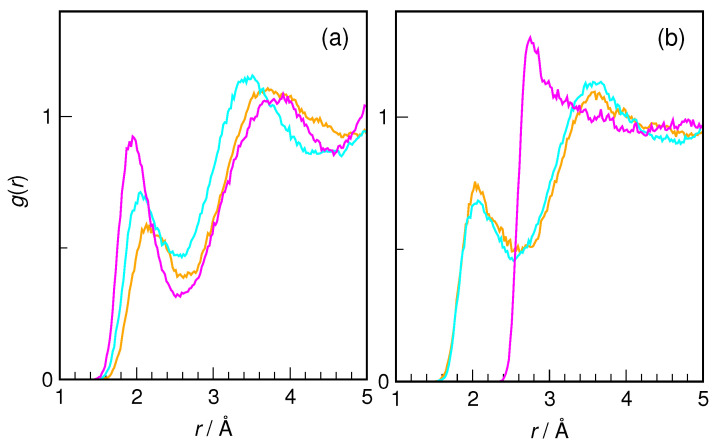
Radial distribution functions for H_5_···O*_w_* (orange), H_6_···O*_w_* (cyan), and H_7_···O*_w_* (magenta) pairs in (**a**) *E*-hydroxyurea and (**b**) *Z*-hydroxyurea.

Urea also forms H-bonds by donating amide protons to water, although they are weaker than the H-bonds to carbonyl oxygen [6]. As can be seen in Figure 8, the same conclusion can be reached for hydroxyurea. Although the most probable distances differ between the three discussed H atoms, the integration of the first peak in RDF gives a similar coordination number of 0.8–1.0. The shortest hydrogen bond is formed by the H_5_···O*_w_* pair in *Z*-HU, for which the maximum of the first peak is at 2.03 Å and the integral is exactly 1.0, indicating a stable H-bond. In other cases, the integration result is slightly less than one. A very special case is presented by a H_7_···O*_w_* pair in *Z*-HU, where it can be concluded that no H-bond is formed with water. This most probably results from the withdrawal of the electron density on nitrogen and the decreased polarity of the N_1_-H_7_ bond.

**Figure 9:**
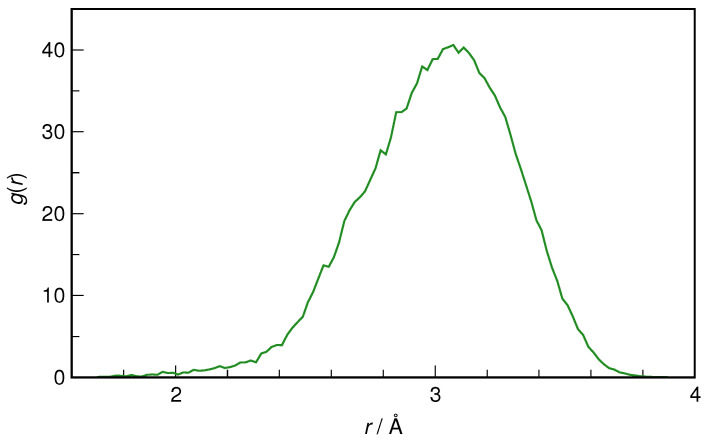
Intramolecular radial distribution function for O_4_···H_9_ pair in *Z*-hydroxyurea.

To stress the crucial absence of intramolecular H-bonds in *Z*-HU found with WT-MTD, the equilibrium AIMD simulations were also checked for their possible signatures (cf. Figure 9). However, the intramolecular distribution of the O_4_···H_9_ distance shows that the two atoms rarely ever come closer than 2 Å, while the most probable distance is 3.1 Å, confirming that the presence of this H-bond may be ruled out with certainty in an aqueous solution, in contrast to the gas phase molecule, where this interaction does indeed stabilize *Z*-HU [26,27,28].

**Figure 10:**
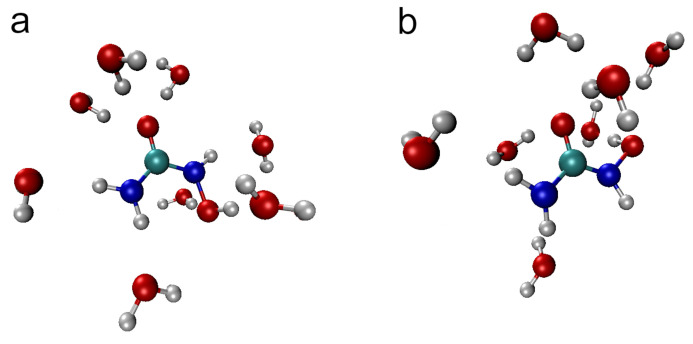
Instantaneous snapshot of the hydration shell of (**a**) *E*-hydroxyurea and (**b**) *Z*-hydroxyurea. Atoms are colored as follows: C (cyan), O (red), N (blue), and H (gray).

In order to summarize the discussion of structural aspects of HU hydration, representative snapshots from the AIMD trajectory of *E*-HU and *Z*-HU with the closest hydrogen-bonded water molecules are shown in Figure 10. The common features of the hydration shell include the relatively short H_9_···O*_w_* hydrogen bond, ca. three hydrogen bonds from water to the carbonyl oxygen, the hydrogen bond donated by water to O_8_, and slightly longer H_5_···O*_w_* and H_6_···O*_w_* hydrogen bonds. Most notable is the presence of a weak H_7_···O*_w_* hydrogen bond in *E*-HU versus its unusual absence in *Z*-HU (cf. Figure 8). Both conformers are also characterized by an instantaneous value of the ω dihedral angle close to 100°, i.e., with the O_8_-H*_w_* bond roughly perpendicular to the plane defined by the OCNO atoms of HU; see Figure 3.

**Figure 11:**
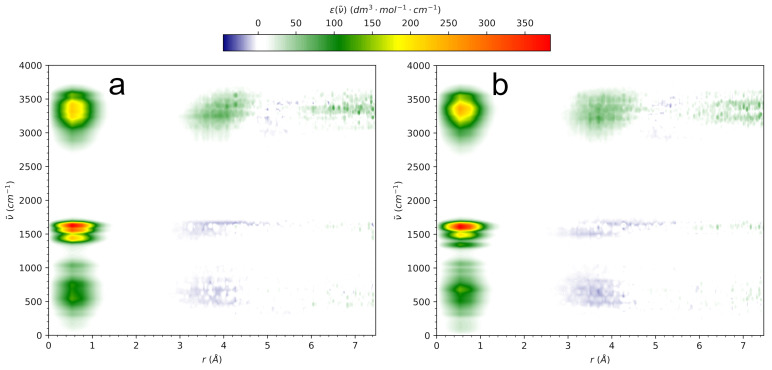
The radially resolved IR spectrum of aqueous (**a**) *E*-hydroxyurea and (**b**) *Z*-hydroxyurea. The center of mass of HU molecule is located at r=0. The local spectral intensity is indicated by color scale.

The influence on water of small solutes, such as osmolytes, is optimally studied with infrared spectroscopy [6,58]. The clear advantage of computational IR spectroscopy over the experiment is the possibility to dissect the IR spectrum of the system into inter- and intramolecular contributions, as well as to spatially decompose the spectra in order to pinpoint the spatial–temporal correlations between IR excitations in different molecules [36,37,38]. The radially resolved IR spectrum gives an unprecedented insight into the interaction of the central solute molecule with the surrounding H-bond network of water [37]. Such spectra for both HU conformers are shown in Figure 11.

The spectral intensity below 1 Å reflects the IR spectrum of the solute, while the cross-correlations at progressively increasing *r* indicate the interactions of HU with its hydration shells. It is obvious that the central dipole effectively couples to the neighboring water molecules in the range of the OH stretching vibrations (3000–3700 cm^−1^), as evidenced by the positive spectral intensity in the range 3–5 Å, roughly the extent of the first hydration shell of HU. This effect extends further to the second hydration shell, as seen by the slightly weaker positive cross-correlation at 6 Å and beyond. Thus, HU effectively couples to the water–H-bonded network and does not disrupt it to a significant extent. It is worth noting that this behavior is shared by aqueous halide anions [37,39] that also show enhancement of the absorption intensity in the same spectral range.

Due to the mutual polarization effects in solution, the spectrum at r→0 is not exclusively due to the solute. It is well known that “solute” spectra in water also contain the contribution of the water IR spectrum [58]. This remains true even for monatomic anions that do not show any of their own IR spectra [37,39]. However, the intense intramolecular modes of HU below 1700 cm^−1^ dominate the spectral response at low *r* values. The latter are effectively quenched by the surrounding water, as evidenced by the clearly negative cross-correlations observed within the first hydration shell.

The two HU conformers show very similar radially resolved IR spectra, indicating that the incorporation of HU into the H-bonded network of water is efficient regardless of its internal conformation. It does seem, though, that *Z*-HU shows slightly better vibrational coupling to the first hydration shell, as indicated by the size and intensity of the respective spectral signal.

**Figure 12:**
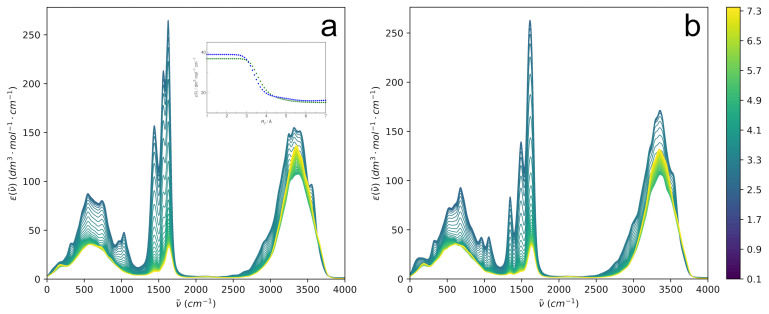
The distance-dependent IR spectrum of aqueous (**a**) *E*-hydroxyurea and (**b**) *Z*-hydroxyurea. The center of mass of HU molecule is located at $R_{c}=0$. The cutoff distance in Å is indicated by color scale. The inset shows the modulation of the distance-dependent spectra at a probing frequency 320 cm^−1^ in *E*-hydroxyurea (green) and *Z*-hydroxyurea (blue).

The IR spectrum of the solute–solvent system may be decomposed in a completely different manner by considering the spectrum of a sphere around the solute containing solvent molecules within the selected cutoff distance. The sphere is smoothly carved out of the system, so the internal dipole moment changes upon departure of the solvent molecules in a continuous manner rather than abruptly. Such distance-dependent IR spectra for aqueous HU are shown in Figure 12.

The most apparent feature of the examined spectra is the expected dominance of the intramolecular spectrum of HU at a low $R_{c}$. However, as more and more solvent molecules are incorporated, the spectra at increasing $R_{c}$ progressively lose their intramolecular character, mostly due to a simple dilution effect, and at $R_{c}\to L$, the IR spectrum tends to the spectrum of bulk water. Although the mid-IR bands of HU are slightly different for the two isomers, their modulation with an increasing cutoff distance looks very similar.

In the search for possible differences between the two isomers, the modulation of the HU band at ∼320 cm^−1^ is examined in detail; see the inset in Figure 12. The choice of the fundamental band is arbitrary to some extent because the modulation with increasing $R_{c}$ looks qualitatively similar for other bands. The band is convenient due to having the same position for both conformers. The intensity is seen to change smoothly from the solute regime at $R_{c}<3$ Å to the bulk solution regime at $R_{c}>5$ Å. The cutoff radius at the inflection point of the intensity modulation curves is selected as characterizing the sphere, as it represents the most tightly bound water molecules that are strongly vibrationally coupled to the solute. Such a procedure gives interesting insight into the solute-affected IR spectrum of aqueous systems, as discussed previously [58]. The selected cutoff radii minimally differ for the two solutes, being 3.7 Å for *E*-HU and 3.5 Å for *Z*-HU.

**Figure 13:**
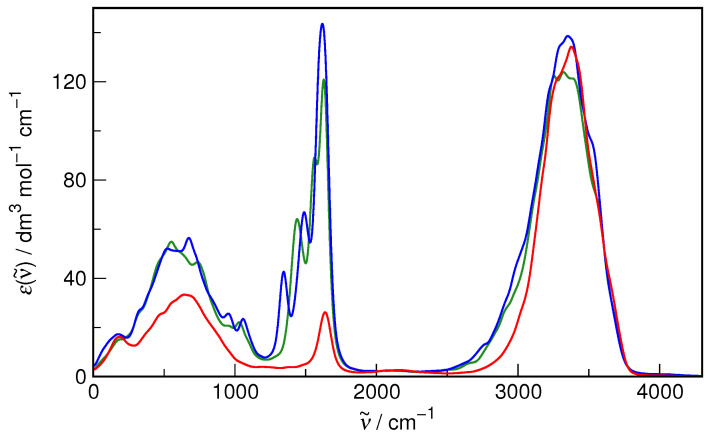
The distance-dependent IR spectrum of aqueous *E*-hydroxyurea at $R_{c}=3.7$ Å (green) and *Z*-hydroxyurea at $R_{c}=3.5$ Å (blue), compared with the bulk water IR spectrum (red).

Most interesting for characterizing the relative influence of the solute on the solvent is the average number (possibly fractional) of H_2_O molecules contained within the selected radius. It is found here that it is appreciably higher for *E*-HU (3.4) than for *Z*-HU (2.5), indicating that *E*-HU spectrally influences more neighboring waters. However, as seen in Figure 13, where the spectra at the selected cutoff radii are compared with the bulk water spectrum, the influence of both conformers on the OH stretching band of water is very similar. Most notably, both *E*-HU and *Z*-HU, when compared to bulk water, do not influence the high wavenumber side of the OH stretch. On the contrary, the low wavenumber side of this band has visibly raised intensity for both conformers. Since the red shift of the OH stretching band signifies the strengthening of the water H-bond network, it is perhaps the ultimate proof that both *E*-HU and *Z*-HU have a profound enhancing effect on this network and should be treated as strongly hydrated solutes, as inferred from volumetric studies [32].

The energetic impact of HU on the hydrogen bond network of water may be estimated based on a correlation between hydrogen bond enthalpy and the red shift of the OH stretching vibration band,

(9) $$-\Delta H=1.3\;{\left(\Delta \tilde{\nu }\right)}^{1/2}\;,$$

where $\Delta \tilde{\nu }=3660-\tilde{\nu }$ is the magnitude of the red shift from the reference value of the “free” OH group for water diluted in CCl_4_ [59]. Based on the distance-dependent spectra shown in Figure 13, the computed red shifts are 283 cm^−1^, 309 cm^−1^, and 338 cm^−1^ for bulk water, hydrated *Z*-HU, and hydrated *E*-HU, respectively. These values directly correspond to an increase in hydrogen bond enthalpy—with respect to the value in bulk water—by ca. 1 kJ·mol^−1^ and 2 kJ·mol^−1^ (per bond) for hydrated *Z*-HU and hydrated *E*-HU, respectively. This leads to the conclusion that, paradoxically, even though *Z*-HU is the more favorable conformer in an aqueous solution based on the free energy estimation, the *E*-HU conformer shows a slightly better effect on the enthalpy of the nearest hydrogen bonds.

Unfortunately, no experimental data on the hydration of HU studied by IR spectroscopy are currently available. The application of difference spectroscopy techniques, which are most successful in delivering invaluable data on electrolyte and non-electrolyte hydration [2,3,49], in the particular case of HU would be hampered by the presence of OH and NH groups in the structure of the solute that absorb in the same spectral range as the OH stretching vibration of H_2_O. However, we envisage a possible pathway to obtaining the spectra of water affected by HU by using sophisticated spectral analysis techniques based on chemometry [60], which may be the direction of future experimental work on HU hydration.

## 4. Computational Methods

AIMD simulations [33] were performed using the cp2k 6.0 suite of programs [61,62,63]. The electronic structure of the system was represented in terms of DFT [34,35]. The revPBE exchange-correlation functional [40,41] from the generalized gradient approximation (GGA) family and available in the libXC library [64] was used, as justified above. Cp2k utilizes double basis set expansion for valence electrons in terms of Gaussian atomic orbitals and plane waves (GPW representation) [65]. Here, the TZV2P basis set for atomic orbitals was used, which is of triple-ζ quality with a double set of polarization functions and is a de facto standard in AIMD simulations of liquid water [36,37,42,53,54]. The cutoff for the auxiliary plane wave expansion of the electron density was set to 500 Ry. In contrast to explicitly treated valence electrons, core electrons were represented by norm-conserving GTH pseudopotentials (PPs) [66], and the generic PPs parameterized for the PBE functional were applied. As reported previously, using the PBE PPs in combination with revPBE explicit orbitals gives essentially the same results as using reoptimized PPs [67]. Finally, the electron density and its derivative were smoothed on a spatial integration grid (keywords XC_SMOOTH_RHO NN50 and XC_DERIV NN50_SMOOTH in cp2k) since this procedure was previously found to improve the description of the potential energy surface of the water dimer [68]. The DFT-D3 empirical dispersion correction [69], including only two-body terms without damping and a cutoff set to 15 Å, was applied on top of the DFT energy.

The numerical evaluation of the Kohn–Sham equations and the nuclear propagation on the Born–Oppenheimer surface were performed using the Quickstep electronic structure module available in cp2k [70]. The full diagonalization of the Kohn–Sham matrix was avoided by using the efficient orbital transformation method with a diagonalization-based preconditioner [71].

Herein, the results of AIMD simulations of three systems are reported. The hydration of both the *E* and *Z* conformers of hydroxyurea (*E*-HU and *Z*-HU, see Figure 1) was studied independently. The two most stable conformers were selected based on previous gas phase quantum chemical (MP2 level) as well as DFT studies [26,27,28,29], and the initial configurations of both HU isomers were taken from Ref. [26]. The HU–water systems consisted of a single HU molecule solvated by 109 H_2_O molecules in a cubic simulation cell with applied periodic boundary conditions, corresponding to a molality of HU ∼0.51 mol·kg^−1^. The size of the cell (L≈14.97 Å) was based on interpolating the experimental density of aqueous HU solutions [32]. Additionally, liquid water was studied as a reference system. The cubic simulation cell contained 128 H_2_O molecules in a volume corresponding to the experimental density of water at 298.15 K (L≈15.66 Å) [72].

Each system was initially equilibrated for at least 40 ps in the canonical (NVT) ensemble with the temperature set to 298.15 K and stabilized by the CSVR thermostat [73]. The time constant for the latter was set to 2000 cm^−1^ (≈16.67 fs). The simulation time step was 0.5 fs. After the equilibration period, the canonical trajectory was continued, and a set of 16 initial conditions separated by 2–3 ps was sampled from it. These were used to initialize production simulations in the microcanonical (NVE) ensemble, lasting 20 ps each. In these runs, the centers of maximally localized Wannier functions (MLWFs) [48] were computed every 2 fs and stored along with atomic positions and velocities. All reported observables were averaged over the 16 trajectories, thus providing canonical averages.

Classical molecular dipole moments were obtained at each saved trajectory frame by summing over positive nuclei and negative MLWF centers belonging to a molecule. IR spectra were calculated from Fourier transforms of time correlation functions of finite differences in dipole moments [37,47]. In order to aid their interpretation, dipolar decomposition schemes for solute–solvent systems introduced previously by one of the authors were used; see above for details. The spectral resolution was set to 1 cm^−1^, and all IR spectra were smoothed by a 20 cm^−1^-width Gaussian filter. The refractive index contributions to the IR spectra were removed using the numerical Kramers–Kronig transform [37], taking the experimental optical refractive index of H_2_O, $n_{D}=1.3325$ [74].

In order to check the potential energy surface (PES) for interconversion between *E*-HU and *Z*-HU, well-tempered metadynamics (WT-MTD) simulations were additionally performed [50]. The OCNO dihedral angle in HU was selected as the relevant collective variable (CV). Two independent WT-MTD runs were initiated, starting from the *E*-HU or *Z*-HU conformer. The initial configurations for these runs were sampled from the equilibrium canonical simulations described above. The Gaussian hills were characterized by a height and width equal to 0.001 and 0.02 Hartree, respectively, and were deposited every 50 WT-MTD steps (i.e., every 25 fs). The WT-MTD sampling temperature was set to 1500 K. The PES for the OH group rotation in HU was also sampled separately for each conformer by choosing the CNOH dihedral angle as the CV, using the same WT-MTD parameters as above.

The total trajectory time for AIMD simulations reported in this work is ca. 1.3 ns (2.6·10^6^ frames), corresponding to ca. 300 days of computer wall time.

## 5. Conclusions

Hydration, as an important physicochemical phenomenon, has been extensively studied for decades. *N*-hydroxyurea, a widely used chemotherapeutic agent, has thus far never been comprehensively studied with respect to its interactions with water in an aqueous solution. In this work, we conclusively demonstrate its strong interaction with the first solvation shell by exploring the radial distribution functions around different functional groups of the HU molecule and arriving at the picture of a tightly bound solvation complex. The average coordination numbers are 1.0 for the H_9_···O*_w_* interaction and ca. 2.5 for O_4_···H*_w_* interaction. HU also forms, on average, 0.8–1.0 contacts with the water oxygen via its amine H atoms, with the notable exception of the H_7_···O*_w_* hydrogen bond, which is absent for *Z*-HU. The radially resolved IR spectra prove that HU effectively couples with the OH stretching vibration of liquid water in the range 3000–3700 cm^−1^ and over an extended spatial region covering up to its second hydration shell, thus being effortlessly incorporated in its hydrogen bond network. The analysis of distance-dependent IR spectra of hydrated HU shows that it strongly spectrally affects the nearest 3.4 and 2.5 H_2_O molecules for *E*-HU and *Z*-HU, respectively, in spheres with cutoff radii of 3.7 Å and 3.5 Å, respectively. The obtained spectra additionally demonstrate the low wavenumber broadening of the OH stretch, which is known to reflect the general strengthening of the hydrogen bond energy, leading to the conclusion that HU behaves as a structure-making molecule. This is further corroborated by the increase in the average hydrogen bond enthalpy by 2 kJ·mol^−1^ and 1 kJ·mol^−1^ (per bond) for *E*-HU and *Z*-HU, respectively, based on the empirical correlation of the OH stretching band red shift with enthalpy. Exploration of the free energy surface for conformer interconversion with metadynamics shows a surprising preference for the *Z*-HU conformer (with OCNO dihedral angle approx. 10°), which contradicts previous gas phase studies that unanimously point to the *E*-HU (OCNO dihedral angle close to 160°) being the more stable one. Thus, the solute–water interactions are shown to dramatically influence the conformational equilibrium of HU. The ΔF value for *E*-HU ⇄ *Z*-HU interconversion is 17.4 kJ·mol^−1^, with a sizable free energy barrier separating the two conformers and reaching ∼45 kJ·mol^−1^ in the *Z*-HU → *E*-HU direction and ∼28 kJ·mol^−1^ in the opposite direction. The free energy surface for the CNOH dihedral angle in HU shows that its preferred value is close to 90–100°, i.e., perpendicular to the plane defined by the OCNO atoms. The results obtained here are important for future studies of, e.g., HU transport and docking, as knowledge of hydration structure is necessary to describe the in vivo properties of the molecule in the cytoplasmic environment. In particular, the easy incorporation of HU into the hydrogen bond network of water seems to have far-reaching implications for its mechanism of action in ribonucleotide reductase, where the displacement of strongly bound water molecules at the catalytic radical site is the prerequisite for the further binding and action of hydroxyurea.

## Data Availability

The data presented in this study are available on request from the corresponding author.
